# Retraction: miR-5089-5p suppresses castration-resistant prostate cancer resistance to enzalutamide and metastasis via miR-5089-5p/SPINK1/ MAPK/MMP9 signaling

**DOI:** 10.18632/aging.104212

**Published:** 2020-10-31

**Authors:** Zhi-Chao Wang, Yan Li, Ke-Liang Wang, Lu Wang, Bo-Sen You, Dan-Feng Zhao, Zhong- Qing Liu, Rui-Zhe Fang, Jia-Qi Wang, Wei Zhang, Jin-Ming Zhang, Wan-Hai Xu

**Affiliations:** 1 Heilongjiang Key Laboratory of Scientific Research in Urology, The Fourth Affiliated Hospital of Harbin Medical University, Harbin 150001, China; 2 Department of Anesthesia, The Fourth Affiliated Hospital of Harbin Medical University, Harbin 150001, China

**This article has been retracted:** Authors concluded that the experimental results are not solid enough to support their conclusions. Thus, they have made the decision to retract the article upon the agreement of all listed authors.

Original article: Aging. 2020; 12:14418–14433.  . https://doi.org/10.18632/aging.103485

